# App-based intervention for reducing depressive symptoms in postpartum women: Protocol for a feasibility randomized controlled trial

**DOI:** 10.1016/j.invent.2023.100616

**Published:** 2023-03-31

**Authors:** Pamela Franco, Marcia Olhaberry, Pim Cuijpers, Saskia Kelders, Antonia Muzard

**Affiliations:** a Doctoral Program in Psychotherapy, School of Psychology, Pontificia Universidad Católica de Chile, Av. Vicuña Mackenna 4860, Santiago, Chile; b Millennium Institute for Research in Depression and Personality (MIDAP), Santiago, Chile; c School of Psychology, Pontificia Universidad Católica de Chile, Av. Vicuña Mackenna 4860, Santiago, Chile; d Department of Clinical, Neuro and Developmental Psychology, Amsterdam Public Health Research Institute, Vrije Universiteit Amsterdam, De Boelelaan 1105, 1081 HV Amsterdam, the Netherlands; e Centre for eHealth & Wellbeing Research, Psychology, Health & Technology, Faculty of Behavioral, Management and Social Sciences, University of Twente, Drienerlolaan 5, 7522 NB Enschede, the Netherlands; f Optentia Research Unit, North-West University, VTC, South Africa; g School of Psychology, Finis Terrae University, Chile

**Keywords:** Randomized controlled trial, Internet, Guided self-help, Depression, Postpartum, Cognitive-behavioral therapy, Feasibility, Acceptability

## Abstract

**Background:**

Chile has a high prevalence of postpartum depression and a significant treatment gap. Some barriers to postpartum depression care uncover the need for more easily accessible and lower-cost interventions. Chile's high utilization of digital technologies across all social strata and the increased use of pregnancy and parenting apps open the possibility of delivering interventions through mobile devices. Cognitive-behavioral internet-based interventions have proven to be effective in reducing symptoms of depression in high-income countries. However, in Chile, this is an underdeveloped field. This manuscript describes a randomized controlled trial protocol that will examine the feasibility and acceptability of a guided 8-week cognitive-behavioral app-based intervention for Chilean postpartum women with depressive symptoms.

**Method:**

A small-scale parallel 2-arms trial will be conducted. Postpartum women with minor or major depression will be randomized to the app-based intervention or waitlist. The primary outcomes are feasibility and acceptability variables, mainly; recruitment and eligibility rates, intervention and study adherence, and participants' intervention satisfaction, use, and engagement. Semi-structured interviews with a sub-sample will provide more information about the participants' experience with the intervention. Women's depression status will be assessed at pre-treatment, post-treatment, and 1-month follow-up. Other secondary outcomes will include participants' perceived social support, mother-infant bonding, and maternal satisfaction and self-efficacy.

**Discussion:**

This will be the first internet-based intervention aimed at reducing postpartum depression symptoms developed and studied in Chile. If the intervention and procedures prove feasible and acceptable, we plan to study its efficacy in a definitive controlled trial. If the intervention demonstrates to be effective, the aim is to implement it within the Chilean healthcare setting.

## Highlights

• Chile has a high prevalence of postpartum depression and a significant treatment gap.
• Online interventions may overcome barriers to postpartum depression care.
• The feasibility of an intervention for postpartum depressive symptoms will be assessed.

## 1 Background

Maternal mental health problems pose significant global public health challenges (Hahn-Holbrook et al., 2018). Among these problems, postpartum depression (PPD) is the most common condition (Bydlowski, 2015). A meta-analysis of 291 studies using the Edinburgh Postnatal Depression Scale (EPDS) on 296.284 women from 56 countries found a global prevalence of PPD of 16.7 %, with significant heterogeneity across nations, ranging from 3 % (2–5 %) in Singapore to 38 % (35–41 %) in Chile (Hahn-Holbrook et al., 2018).

PPD is a devastating mental illness because it affects the whole family (Letourneau et al., 2012); it is associated with overall poor maternal health and impaired maternal functioning (O'Hara and McCabe, 2013; Gjerdingen and Yawn, 2007; Slomian et al., 2019). Impairments in maternal functioning are associated with difficulties in mother-to-infant bonding that may reduce the quality of the mother-infant relationship, which is critical for infant development and mental health (Stein et al., 2014; Slomian et al., 2019). Children of mothers with depression show a higher risk of developing insecure and disorganized attachments (Hayes et al., 2013) and a higher risk of developing depression throughout life (Pawlby et al., 2009). Evidence shows that the negative consequences in the quality of the mother-infant interaction are present even when the depressive symptomatology is subclinical (Tronick and Reck, 2009; Weinberg et al., 2001).

Psychotherapy is recommended as the first-line approach for both antenatal and postpartum depression (perinatal depression, hereafter: PND). It may be combined with pharmacotherapy, depending on depression severity, available options, and women's acceptance (Molenaar et al., 2018). In Chile, two studies have assessed interventions for PPD treatment (Olhaberry et al., 2015; Rojas et al., 2007). Rojas et al. (2007) group psychoeducational intervention showed significant improvement in low-income mothers with PPD compared with those in the usual care group. However, the attendance to the group sessions was meager; the mean number of group sessions attended was 2.7 out of 8, even though the research team offered a person to look after the participant's child during the sessions. The impression of the group coaches was that women liked attending groups, but too many pressing daily difficulties competed with their attendance (Rojas et al., 2007). Olhaberry et al. (2015) intervention consisted of a 4-session video-feedback intervention for mother-baby dyads on women with different levels of depressive symptomatology (from mild to severe). They found a significant increase in maternal sensitivity but no reduction in depressive symptoms.

In Chile, some advances in public policies have sought to improve mothers' and babies' wellbeing. In 2011, maternity leave was extended from 12 to 24 weeks. Additionally, since 2014, all women attending public healthcare facilities are screened for perinatal depression with the EPDS (twice during pregnancy and twice postpartum). However, despite the efforts to apply a protocol for universal PPD screening, early detection, and referral to mental healthcare services (Ministerio de Salud, 2014), there is still a significant treatment gap (Rojas et al., 2018). Barriers to addressing PPD have been identified on different levels (Dennis and Chung-Lee, 2006; Goodman, 2009; Byatt et al., 2012; Byatt et al., 2013; Rojas et al., 2015). At the patients' level, they include misunderstandings or negative beliefs about PPD and psychological treatments, stigma, and fear of being judged as an unfit parent, concerns about the risk of antidepressant use, lack of time or energy, lack of social support, childcare issues, lack of confidence on healthcare professionals and economic barriers. At the perinatal healthcare professionals' level, barriers include a lack of time for further psychological assessment and adequately communicating the diagnostic suspicion during check-ups, in addition to a lack of training in mental health.

In Chile, patients diagnosed with depression by a general practitioner have guaranteed access to treatment at low or no cost. Treatment varies according to depression severity, with group or individual therapy, alone or combined with pharmacotherapy. Nonetheless, public health providers' barriers to mental health care include long waiting lists, a frequency and duration of sessions below international recommendations, and a high rotation of professionals (Rojas et al., 2015; De la Parra et al., 2019). There is, therefore, an urgent need for user-friendly, easily accessible, low-cost, evidence-based psychological interventions to reduce the burden of PPD (Lee et al., 2016).

Nowadays, women are increasingly using the internet as a source of health information during pregnancy and postpartum, regardless of their socioeconomic status (Guerra-Reyes et al., 2016; Slomian et al., 2017). The widespread ownership of mobile phones globally has also prompted attention from the health field on the potential of delivering information and interventions through smartphones (Lal, 2019). Apps are particularly valued because of their convenience, immediacy, and ease of access (Lee et al., 2016). Women around the globe (including Chilean women) are engaging with pregnancy and parenting apps, which are almost becoming a routine part of the maternity experience (Hughson et al., 2018; Franco et al., 2023). This growing use of internet resources for health information is aligned with a trend in the world today: people are more in the lead of their healthcare, and this requires innovative ways of support (van Gemert-Pijnen et al., 2018). Thus, the internet and digital technologies have the potential to reach an enormous number of women who are already seeking information online.

Internet-based psychological interventions have the potential to overcome logistical and financial barriers both on the part of healthcare providers and patients, holding promise for cost-effective interventions (Krausz et al., 2019). Over the past decade, this field has grown in high-income countries (Lal, 2019) and shows a slow but progressive presence in other countries like Chile (Jiménez-Molina et al., 2019; Rojas et al., 2019). Advances in Chile have been facilitated by the fact that digital technologies are now highly utilized across all social strata (SUBTEL, 2021; SUBTEL, 2022). On the other hand, the growth of this field in the country is especially relevant for low-resource health services located in rural, hard-to-reach areas with no specialized mental health care nearby (Rojas et al., 2019).

Internet-based interventions have proven to be effective. Karyotaki et al.'s (2021) conducted an ‘individual participant data’ network meta-analysis that included 39 randomized controlled trials on internet-based interventions for depression (including PND). The meta-analysis demonstrated that both guided (including some form therapeutic support, either synchronous or asynchronous, delivered by a professional or a paraprofessional) and unguided (self-help or with automated support) interventions are associated with a greater reduction in depressive symptoms than treatment-as-usual and waitlist at post-treatment. Differences are maintained at six months and 12 months following randomization. Regarding internet-based interventions exclusively for PND, preliminary findings suggest that they may effectively reduce depressive symptomatology in perinatal women. Mu et al.'s (2021) meta-analysis showed that internet-based interventions could decrease the prevalence of perinatal depression and alleviate depressive symptoms in perinatal women (overall effect size of d = 0.64).

Although internet-based interventions for PND show promising results, they face adherence problems even when the intervention is guided (O'Mahen et al., 2013a; O'Mahen et al., 2013b; Lee et al., 2016; Lau et al., 2017; Forsell et al., 2017). One issue identified to work on is how to appropriately tailor the internet-based intervention's design to ensure usability, acceptability, and engagement among target users while retaining clinical effectiveness (Nicholas et al., 2017). Recent research on intervention development suggests integrating theory- and expert-based approaches with other methods that promote the involvement of the user (and other possible key stakeholders) from an early stage of intervention development and throughout the different stages of the process (van Gemert-Pijnen et al., 2011). The same has been suggested by studies that have assessed mothers' use and preferences for internet-based interventions or resources (Walker et al., 2017; Guerra-Reyes et al., 2016; Ramphos et al., 2019).

### 1.1 Aims of the study

We developed *“Mamá, te entiendo” (“Mom, I get you”),* a guided 8-week cognitive-behavioral app-based intervention. The development of the intervention followed the “CeHRes Roadmap” (van Gemert-Pijnen et al., 2011), a multidisciplinary framework for guiding the development and assessment of eHealth derived from evidence-based strategies (including designing from user-based and persuasive approaches). This study aims to evaluate the feasibility and acceptability of “*Mamá, te entiendo*” through a small-scale randomized controlled trial. This intervention targets postpartum women with minor and major depression. Its aims are reducing depressive symptoms in the general sample and preventing major depression onset in women with minor depression. The feasibility outcomes will provide data to estimate the parameters required to design a definitive randomized controlled trial for assessing intervention efficacy (Arain et al., 2010). A qualitative process evaluation will be included to gain insight into participants' perspectives on the study procedures and intervention.

## 2 Methods and design

### 2.1 Study design

**Fig. 1:**
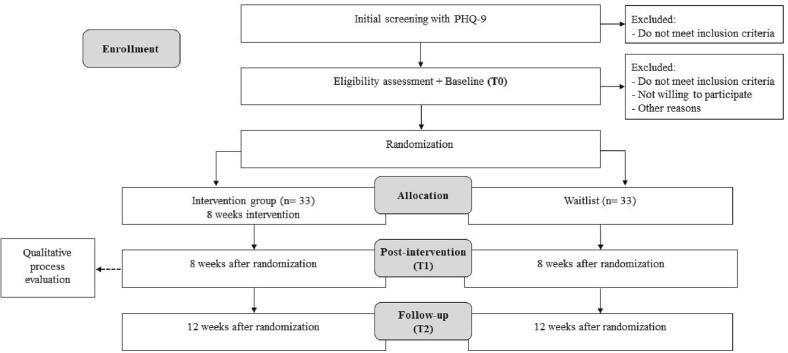
Study flow.

This is a mixed-methods feasibility and acceptability study of an app-based intervention for reducing postpartum depression symptoms. A two-arm, small-scale randomized controlled trial will be conducted with postpartum women with minor or major depression. Women will be randomly assigned to the intervention group (internet-based intervention: “*Mamá, te entiendo*”) or the control group (waitlist: WL). The three primary data collection points for study participants in the intervention group and WL group are as follows: baseline (pre-treatment: T0), post-treatment (8 weeks from randomization: T1), and 1-month follow-up (12 weeks from randomization: T2). See Fig. 1 for a detailed overview of assessment points. All assessments will be administered online through video calls (interviews) and web platforms (self-report questionnaires). A qualitative evaluation will be conducted to have more knowledge about participants' perspectives on the intervention and study procedures. The study has been registered in Clinicaltrials.gov (https://clinicaltrials.gov/) as NCT05643898 (December 2022) and received ethical approval from the Health Sciences Ethical Committee of Pontificia Universidad Católica (Santiago, Chile; protocol ID 210824004).

### 2.2 Participants

Women will be included if they: have a baby between 1 and 7 months of age, have a PHQ-9 (Kroenke et al., 2001; Diez-Quevedo et al., 2001) score ≥ 5 points, have minor or major depression according to the Mini-International Neuropsychiatric Interview (MINI: Sheehan et al., 1998; Ferrando et al., 2000), are at least 18 years old, are Chilean residents, are fluent in written and spoken Spanish, self-reported ownership of a smartphone, and self-reported regular access to the internet. Minor depression will be defined as the presence of two to four concurrent symptoms of depressive disorder (one of which is depressed mood or anhedonia) present most or all the time for at least two weeks, with symptoms causing dysfunction and negative impact on the individual's life (American Psychiatric Association, 2013). Major depression will be defined as the presence of five or more concurrent symptoms of depressive disorder (one of which is depressed mood or anhedonia) present most or all the time for at least two weeks, with symptoms causing dysfunction and a negative impact on the individual's life (American Psychiatric Association, 2013). Participants' exclusion criteria will be the following: illiteracy, moderate or high risk of suicide, history of alcohol or substance abuse since they found out about the pregnancy to date, lifetime history of bipolar disorder or psychotic disorder, and current psychotic features.

### 2.3 Sample size

As feasibility studies are not expected to test statistical significance, the usual power calculation for clinical trials is not normally undertaken (Arain et al., 2010). However, some authors suggest using a sample size of 25 per group for pilot studies (Whitehead et al., 2016). Based on these recommendations and anticipating a possible dropout of 30 %, a sample size of 33 participants per group was set.

### 2.4 Recruitment & screening for eligibility

Recruitment will be conducted online through advertisements on social media platforms. The ads will include general information on the study aims, inclusion criteria, and procedures, and will be linked to a web-based depression symptomatology assessment. Potential participants will be contacted for further eligibility assessment. No incentives for participation will be provided so that the endpoint of participant acceptability is not confounded.

#### 2.4.1 Initial screening

The web-based initial screening will include brief information about the study, informed consent for this assessment, and the Patient Health Questionnaire -9 (PHQ-9: Kroenke et al., 2001; Diez-Quevedo et al., 2001) with automated feedback based on the obtained score. The automated feedback will function with the established score ranges of the scale: minimum, mild, moderate, and severe. Women will be encouraged to discuss their symptoms with their general practitioner or mental health specialist when there is a result of moderate or severe depression or a score of >1 point in question 9 (“Over the last 2 weeks, how often have you been bothered by thoughts that you would be better off dead or of hurting yourself in some way”; response options Not at all (0), Several days (1), More than half the days (2), and Nearly every day (3)). The feedback for women with suicidal ideation will include a dischargeable document with information on depression and mental health care resources. Women in the mild, moderate, and severe ranges will be informed that they are potentially eligible for the study and that they will be contacted for further assessment. Women in the minimum range or with >1 point in question 9, will be excluded.

In this initial screening, the PHQ-9 was used to assess depressive symptoms instead of the EPDS. This decision was made because we thought that women could be overly familiar with the EPDS. Moreover, as women are universally screened at 2 and 6 months postpartum, they could have answered the EPDS in a healthcare check-up recently.

#### 2.4.2 Structured telephone interview and baseline assessment (T0)

Potentially eligible participants will be contacted by telephone to schedule a video call interview with a clinical psychologist. At the beginning of the video call, women will access a web-based informed consent for participating in the study. The interview will collect sociodemographic and clinical history information and assess for eligibility. The Mini-International Neuropsychiatric Interview (MINI: Sheehan et al., 1998; Ferrando et al., 2000) will be used to assess minor and major depression diagnosis and mental health exclusion criteria. After the interview and during the video call, women satisfying the eligibility criteria will access a web link to complete the baseline questionnaires online.

### 2.5 Participant safety

A standardized operating procedure will be followed in case individuals are defined as showing a moderate or severe risk for suicide. During the initial screening and eligibility assessment, individuals with moderate or severe suicidal ideation will be excluded from the study. They will be asked to discuss their symptoms with a general practitioner or mental health specialist as soon as possible. Additionally, they will receive detailed information about the national program for the treatment of depression and emergency contact numbers. After randomization, during other assessment points, women with >1 point in question 9 of PHQ-9 or >1 point in question 10 of the EPDS (“The thought of harming myself has occurred to me”; response options Yes, quite often (3), Sometimes (2), Hardly ever (1) and Never (0)) will be contacted by telephone. They will receive the same information mentioned above. A similar procedure will be followed if participants included in the trial show symptoms of suicidality throughout the study phase (i.e., by mentioning symptoms of suicidality when contacting the e-coach or when the content of their homework exercises hints at potential suicidal ideation). In such a case, the participant's suicidality risk will be assessed with the suicidality module of the MINI Interview. Participants presenting moderate or severe suicidal ideation will be referred to mental health care, and their suicidal risk will be monitored regularly throughout the study.

### 2.6 Randomization

Women who meet inclusion criteria will be randomized to “*Mamá, te entiendo*” or WL. An independent person not involved in the study will generate the allocation sequence. Stratified randomization will be conducted according to depressive symptom severity (minor or major depression). To generate the random allocation sequence, we will use the software Study Randomizer (2017) using permuted block randomization with block size of 6 in a 1:1 ratio. Researchers will be blind to the randomization process and will be notified through email regarding participant allocation by an independent researcher. Once randomized, participants and research personnel will not be blinded to group condition, except for T1 and T2 interviewers. Following the notification, participants will be contacted by email to inform them of their assigned group (i.e., intervention or WL). The intervention group will receive instructions for registering into the app.

### 2.7 Intervention condition: “*Mamá, te entiendo*” (“*Mom, I get you*”)

Participants in this condition will receive a guided internet-based, cognitive-behavioral, 8-week intervention for postpartum depression symptoms. It also includes elements from attachment and mentalization theories. *“Mamá, te entiendo”* is a web app, a type of app that can be accessed through a web browser. It was developed based on the CeHRes Roadmap (van Gemert-Pijnen et al., 2011) through a participatory process that involved continuous evaluation cycles. Ten focus groups were conducted with perinatal women and healthcare professionals, and five usability interviews with potential end users. Additionally, a pilot with ten postpartum women was conducted to assess the app's proper functioning. The development process will be published elsewhere.

*“Mamá, te entiendo”* consists of the following sections:

#### 2.7.1 Main and optional modules

The intervention consists of six main sequential modules and three optional modules. The main modules address the following: Psychoeducation on depression, How the cognitive-behavioral approach works, Identifying thinking errors, Cognitive restructuring, Problem-solving, and Behavioral activation. The optional modules address: Psychoeducation on anxiety, Exposure strategies, and Communicational Skills. Five case examples of mothers illustrate depressive symptoms and techniques. There is also an introductory module describing the app's functioning (without therapeutic content), and a guide about maintaining changes is displayed when the last main module is completed.

#### 2.7.2 Homework exercises (Workbook)

All modules except the two psychoeducational ones include homework. All homework exercises can be accessed through a specific section in the app called “Workbook”. Participants will receive feedback from an e-coach through the app. They also can contact the e-coach to solve doubts about the app content and exercises. The e-coaches will be psychologists, Ph.D. in Psychotherapy candidates, with a Master's degree in clinical psychology, and training in perinatal mental health. The e-coaching protocol of the Caring Universities Project was adapted for this intervention.

#### 2.7.3 Infographics

Another section of the app includes infographics about other relevant topics: 1) Guide to healing, 2) How to strengthen the support network, 3) Postpartum rage, 4) The postpartum body, 5) Dealing with doubts/comparison/over information, 6) Mentalizing my baby, 7) The couple's relationship in the postpartum period, 8) Returning to work after the maternity leave.

#### 2.7.4 Reading for the support network

A shareable document with reading about how to support a loved one struggling with depression symptoms is included.

#### 2.7.5 Favorites section

Women can tick any part of the content as “add to favorites” and save it in a “Favorites” section.

#### 2.7.6 Resources section

A resources section includes links to websites, hotlines, and documents of interest.

#### 2.7.7 Other sections

The app includes a section presenting the team that contributed to the app's development and the content references. There is a contact section where participants can send a message to the Information and Technology support team to notify them about technical issues if they appear.

Participants are instructed to add a shortcut to “*Mamá, te entiendo*” web app to their smartphone home screen after registration.

### 2.8 WL condition

Participants in the WL condition will receive the same assessments as those in the intervention condition. Participants allocated to WL may access their local mental health services during their waiting period. After the follow-up assessment, the WL group will be offered access to the intervention.

### 2.9 Outcome assessments

#### 2.9.1 Primary outcomes: feasibility and acceptability

Feasibility and acceptability outcomes of interest relate to methodological, procedural, and clinical uncertainties. They will examine recruitment and eligibility; data collection; attrition; participants' intervention adherence, use, and acceptability; and resources needed to complete the study and intervention. Table 1 shows more detail on the feasibility and acceptability outcomes. Intervention credibility and expectancy of benefit will be assessed at baseline by the CEQ-6 (Devilly and Borkovec, 2000), and satisfaction with the intervention will be evaluated post-intervention by the CSQ-8 (Attkisson and Greenfield, 1994; Vázquez et al., 2019). A sub-sample of participants will be selected and invited to participate in a qualitative evaluation to gain more insight into participants' perspectives on the intervention and study procedures.

**Table 1:** Overview of feasibility and acceptability outcomes.

Outcome	Evaluation
Recruitment and eligibility	Number assessed for eligibility by the initial screening
	The proportion of potentially eligible participants from the initial screening
	Number contacted for the eligibility interview
	Number evaluated for eligibility by the eligibility interview
	The proportion fulfilling inclusion criteria (eligibility rate)
	The proportion diagnosed with minor depression
	The proportion diagnosed with major depression
	Reasons for ineligibility
Participants' expectations	Intervention credibility and expectancy of benefit (CEQ-6)
Data collection	Percentage completing assessments at the different time points
Attrition	Rates of study dropout
	Rates of intervention dropout
Participants' adherence to the intervention	Percentage of participants who completed all main modules
	Average, range, and standard deviation of the number of main modules completed
	Average, range, and standard deviation of the number of modules completed
	Average, range, and standard deviation of homework exercises sent to the e-coach
Participants' acceptability of intervention and data collection, and exploration of mechanisms of impact	Satisfaction with the intervention (CSQ-8)
	Engagement with the intervention (TWEETS)
	Impressions and experiences of working with the intervention (positive and negative) and of completing questionnaires and interviews (with a subsample; qualitative)
	Views concerning the impact of the intervention and its different features on their mood
	Reasons for poor attendance and withdrawal (with a subsample; qualitative)
Resources needed to complete the study and the intervention	Length of time required for: ‐ Participants to complete questionnaires and interviews ‐ e-coaches to deliver the intervention ‐ Study personnel to administer the study

CEQ-6: Credibility and Expectancy Questionnaire-6; CSQ-8: Client Satisfaction Questionnaire-8; TWEETS: TWente Engagement with Ehealth Technologies Scale.

##### 2.9.1.1 Credibility expectancy questionnaire (CEQ-6)

The CEQ (Devilly and Borkovec, 2000) is a 6-item self-report questionnaire that measures treatment credibility, defined as “how believable, convincing, and logical the treatment is” and expectancy, defined as “improvements the client believes will be achieved”. The CEQ has good internal consistency and good test-retest reliability (Devilly and Borkovec, 2000).

##### 2.9.1.2 Client satisfaction questionnaire (CSQ-8)

The CSQ-8 (Attkisson and Greenfield, 1994) is an 8-item self-report questionnaire that assesses the general level of satisfaction with a service received. The Spanish version of the CSQ-8 has adequate psychometric properties and maintains those of the original questionnaire (Vázquez et al., 2019).

##### 2.9.1.3 TWente engagement with Ehealth technologies scale (TWEETS)

The TWEETS (Kelders et al., 2020) is a 9-item self-report measure that assesses engagement with an eHealth technology. Of the nine items, three are aimed at assessing behavioral engagement, three at cognitive engagement, and three at affective engagement. Each item is measured on a 5-point Likert scale ranging from “strongly disagree” to “strongly agree”. The TWEETS performs well as an engagement measure with high internal consistency, reasonable test-retest reliability and convergent validity, good divergent validity, and reasonable predictive validity (Kelders et al., 2020). A forward-backward translation process was conducted to produce a Spanish version for this study.

##### 2.9.1.4 Qualitative process evaluation

After the follow-up assessment, a sub-sample of participants from the intervention group will be invited to participate in semi-structured interviews. These participants will be selected through non-randomized propositive sampling according to participants' adherence (high and low), and the sample size cannot be stated a priori. High adherent participants will be defined as completing at least four main modules, and low adherence will be defined as completing less than three. However, these criteria may be adjusted later according to the data. The interviews will explore positive and negative impressions and general experiences with the intervention and study procedures. In addition, to examine possible mechanisms of change in highly adherent participants, their views concerning the impact of the intervention and its different features on their mood and lives more generally will be explored. Interviews with poor adherent participants will explore reasons for disengaging and barriers to treatment and examine suggestions for future intervention development and study procedures.

#### 2.9.2 Secondary outcomes

The secondary outcomes of this study are depression, perceived social support, mother-infant bonding, maternal satisfaction and self-efficacy, and mental health care services use.

##### 2.9.2.1 Mini-international neuropsychiatric interview (MINI)

The MINI (Sheehan et al., 1998; Ferrando et al., 2000) explores 17 mental disorders defined in the DSM-IV-R (axis I), focusing on current symptomatology. This study will use the MINI to assess eligibility and as an outcome measure. It will also evaluate the presence of generalized anxiety disorder. At T1 and T2, only the major depressive episode section will be used. The Spanish version of this instrument has shown acceptable sensitivity and specificity for the most prevalent psychiatric disorder diagnosis (Bobes, 1998). Its psychometric properties have not been studied in the Chilean population, but it has been used in perinatal mental health research (Olhaberry et al., 2015; Rojas et al., 2007). The MINI interviewers will be clinical psychologists and will be masked for the condition.

##### 2.9.2.2 Patient health questionnaire -9 (PHQ-9)

The PHQ-9 (Kroenke et al., 2001; Diez-Quevedo et al., 2001) is a 9-item self-report measure of depressive symptoms experienced over the past two weeks. The PHQ-9 is based on the Diagnostic Statistical Manual, fourth edition (DSM-IV: APA, 2000) diagnostic criteria for major depressive disorder. Participants rate the frequency of symptoms (e.g., ‘Feeling down, depressed, or hopeless’) on a 4-point scale ranging from “not at all” to “nearly every day”. Total scores range from 0 (no symptoms) to 27 (severe). Scores >9 are indicative of a clinically significant level of depressive symptoms. The measure has good psychometric properties (Kroenke et al., 2001) and has been validated in postpartum samples (Gjerdingen et al., 2009). The Spanish version (Diez-Quevedo et al., 2001) has been validated in Chile, showing good psychometric properties (Baader et al., 2012; Saldivia et al., 2019).

##### 2.9.2.3 Edinburgh postpartum depression scale (EPDS)

The EPDS (Cox et al., 1987) consists of ten multiple-choice questions on a 4-point scale ranging from 0 to 3 according to the severity of the symptoms. The total score goes from 0 (no symptoms) to 30 (severe symptoms). It has been validated in various countries and cultures, including Chile (Jadresic et al., 1995). Higher scores indicate more significant depressive symptoms, and three categories of depressive symptomatology are identified: no risk, probable risk, and probable depression.

##### 2.9.2.4 Multidimensional scale of perceived social support (MSPSS)

The MSPSS (Zimet et al., 1988) is a 12-item measure of perceived adequacy of social support from three sources: family, friends, & significant other. It was translated and validated in Chile (Arechabala Mantuliz and Miranda Castillo, 2002). In the Chilean version, the questions are rated on a 4-point scale ranging from “rarely” to “always”.

##### 2.9.2.5 Postpartum bonding questionnaire (PBQ)

The PBQ (Brockington et al., 2006) is a 25-item self-report measure that assesses mother-infant bonding through the mother's feelings and attitudes toward her infant. The questions are rated on a 6-point Likert scale ranging from “not at all” to “always”. A validated Spanish version of the PBQ will be used in this study (Garcia-Esteve et al., 2016).

##### 2.9.2.6 Escala de Evaluación parental [parental assessment scale] (EEP)

The EEP (Farkas-Klein, 2008) is a 10-item self-report measure that assesses satisfaction and self-efficacy regarding motherhood in women with children between 0 and 2 years of age. Each item is measured on a 10-point Likert-type scale, ranging from “totally Disagree” to “totally agree”. The scale was developed and validated in Chile, showing good psychometric properties.

##### 2.9.2.7 Mental healthcare services use

Data on mental healthcare services use (presence/absence, frequency, and duration) will be collected.

#### 2.9.3 Sociodemographic and clinical history variables

Data on age, education, socioeconomic status, employment status, relationship status, ethnicity, nationality, number of children, number of people who comprised the family unit, pregnancy planning, pregnancy complications, baby's date of birth, mother and baby current health problems, prior and current psychological and psychiatric treatment (diagnosis, treatment type, and duration) and the presence of early experiences of sexual abuse and violence will be collected at the eligibility interview. Data on employment and relationship status will be updated at post-intervention and follow-up.

### 2.10 Data collection and management

All data will be collected online via video call (interviews) or web platform (self-report questionnaires). Participants' data that can be directly traced to them (names, email addresses, and telephone numbers) will be coded to protect their privacy. Non-coded data will be saved separately, and only the principal researcher will have access to this data file. Self-report data will be collected using a secured online-based assessment system. All measurements collected during the study with their time points are shown in Table 2. An email account was created for the study to send participants allocation. Only the principal researcher will have access to the study email account. Semi-structured acceptability interviews will be recorded and transcribed. The transcriptions will have no personal data, and the recordings will be deleted after transcription.

**Table 2:** Overview of measures and assessment points.

Variable	Measure	Assessment points		
		T0	T1	T2
Sociodemographic and clinical history variables	Structured questions	x	Update on employment situation & relationship status	Update on employment situation & relationship status
Diagnostic interview	MINI	x	Only depression section	Only depression section
Intervention credibility and expectancy of benefit	CEQ-6	x		
Depression	PHQ-9	x	x	x
Postpartum depression	EPDS	x	x	x
Social support	MSPSS	x		x
Mother-infant bonding	PBS	x		x
Maternal satisfaction and self-efficacy	EEP	x		x
Engagement with the technology	TWEETS		x	
Satisfaction with the intervention	CSQ-8		x	

T0 = pre-treatment (baseline).

T1 = post-intervention (8 weeks after randomization).

T2 = follow-up (12 weeks after randomization).

MINI: Mini-International Neuropsychiatric Interview.

CEQ-6: Credibility and Expectancy Questionnaire-6.

PHQ-9: Patient Health Questionnaire-9.

EPDS: Edinburgh Postnatal Depression Scale.

MSPSS: Multidimensional Scale of Perceived Social Support.

PBS: Postnatal Bonding Scale.

EEP: Escala de Evaluación Parental.

TWEETS: TWente Engagement with Ehealth Technologies Scale.

CSQ-8: Client Satisfaction Questionnaire-8.

Regarding the data stored by the intervention, the web app uses secure HTTPS, which encrypts requests and responses. To register, participants must create a password that will be requested for signing in. A password also protects the admin/coach platform; only the principal researcher and the coaches have this password. The users' and the admin/coach platform's passwords are encrypted. The informed consent form includes information about the collection and management of personal data. More details about the management of the data stored by the app can be found in the Privacy Policy that participants must accept before registering, which they can return to later in the About section.

### 2.11 Analyses

Statistical analysis of feasibility indicators is described in Table 1. The qualitative data from the interviews will be analyzed by two independent coders using thematic content analysis (Braun and Clarke, 2006). Themes will be identified, described, and subsequently categorized. Descriptive statistics will be reported, including the means and standard deviations (SDs) or medians and interquartile ranges for each questionnaire at T0, T1, and T2.

For preliminary efficacy on depressive symptomatology reduction, data will be analyzed following CONSORT guidelines; between-group comparisons will be by intention-to-treat. Missing data will be imputed using the Markov Chain Monte Carlo (MCMC) multivariate imputation algorithm. Comparison between groups will use repeated measures analysis of covariance (ANCOVA) with adjustment for baseline in a random-effects model. Interaction between time and group will be assessed (for changes in group effects with time). In the absence of such an interaction, the overall difference between groups across the two follow-up assessments will be calculated (95 % CIs and *p* values). Secondary analyses will include an adjustment for baseline characteristics. Other secondary outcomes will be analyzed with the same procedures.

## 3 Discussion

“*Mamá, te entiendo*” was developed in response to the high rates of PPD, the barriers to mental health care from both women and health care providers, and the increased use of smartphones and perinatal apps in Chile. Evidence shows that PPD is associated with overall poor maternal (O'Hara and McCabe, 2013; Gjerdingen and Yawn, 2007; Slomian et al., 2019) and infant health (Stein et al., 2014; Slomian et al., 2019; Hayes et al., 2013; Pawlby et al., 2009), even when the symptomatology is subclinical (Tronick and Reck, 2009; Weinberg et al., 2001). Internet-based interventions could alleviate some pressure put on in-person health care services and overcome barriers to help-seeking, providing an alternative or complementary option for widespread perinatal mental health care provision (Spadaro et al., 2022).

The study presented in this protocol will examine the feasibility and acceptability of “*Mamá, te entiendo*” (“Mom, I get you”), a guided cognitive behavioral app-based intervention for reducing postpartum depression symptomatology and preventing the onset of major depression. Assessing the feasibility and acceptability of complex interventions and study procedures are strongly recommended to estimate important parameters and answer key uncertainties required to inform the design of future trials (Eldridge et al., 2016). Given the novelty of the intervention in the country, assessing the acceptability and feasibility of the intervention and study procedures is of great importance in informing intervention refinements and planning a future definitive controlled trial.

One strength of this study is the approach used to develop the intervention, which tries to fulfill the needs of Chilean perinatal women. The development process of the intervention was based on the “CeHRes Roadmap” (van Gemert-Pijnen et al., 2011), which includes the involvement of potential end users and other stakeholders. Also, collecting both quantitative and qualitative data for answering the research questions provides more in-depth findings and can balance out the limitations of each method. On the other hand, some challenges could be expected in this study. Postpartum women will be self-referred to this trial. It is possible that only highly motivated women will be included in our study; thus, the sample will not represent all Chilean women with postpartum depression symptoms.

Regarding the guidance delivered by the e-coach, challenges may arise related to the amount of support provided versus the amount of support expected or needed by the mothers. On the other hand, to assess who would benefit most from the intervention, including more instruments that assess participants' characteristics (like resilience, personality traits, or birth trauma) would have been optimal. However, we tried to keep the number of instruments short not to compromise participants' adherence.

If the intervention and procedures prove feasible and acceptable, the next step will be to study the intervention in a definitive controlled trial. If the intervention demonstrates to be effective, the aim is to implement it within the Chilean healthcare setting.

## Trial status

The trial recruitment will start in March 2023.

## Ethics

Approved 07/10/2021, Pontificia Universidad Católica de Chile Health Sciences Scientific Ethics Committee (Av. Libertador Bernardo O'Higgins 340, Santiago, Chile; +56 22 354 2397; eticadeinvestigacion@uc.cl).

## Funding

This work was supported by the Chilean National Agency of Research and Development (Agencia Nacional de Investigación y Desarrollo; ANID) and the Millennium Institute for Research in Depression and Personality (MIDAP). ANID provided a PhD scholarship (Doctorado Nacional 2019 – 21190745) and research funding for the first author and a PhD scholarship for the fifth author (Doctorado Nacional 2020- 21200074). MIDAP provided research funding for the first author.

## Declaration of competing interest

The authors declare that they have no known competing financial interests or personal relationships that could have appeared to influence the work reported in this paper.
